# 554 Supplemental Nutrition Support in Pediatric Patients with 15-30% TBSA Burn Injuries

**DOI:** 10.1093/jbcr/iraf019.183

**Published:** 2025-04-01

**Authors:** Jennifer Shiel, Christina Sunderman, Kathy Prelack, Elena Smith, Caitlin Phillips, Sara Higginson

**Affiliations:** Shriners Children’s - Boston; Shriners Children’s – Ohio; Shriners Children’s - Boston; Shriners Children’s – Ohio; Shriners Children’s – Ohio; Shriners Children’s – Ohio

## Abstract

**Introduction:**

The post-burn hypermetabolic response can elevate nutritional requirements, rapidly leading to calorie and protein malnutrition. Meeting these accelerated needs orally often challenges patients, warranting supplemental nutrition support. This study evaluated the clinical benefits of adjunctive enteral nutrition in pediatric patients 18 years of age and younger, with 15-30% TBSA burns.

**Methods:**

Five-year multi-center retrospective chart review of patients admitted to two pediatric burn units with 15-30% TBSA burns. Outcome variables included enteral nutrition support and timing, medical length of stay (MLOS), wound length of stay (WLOS), number of surgical procedures, achievement of calorie and protein goals, and anthropometrics changes. Differences between tube-fed (TF) and non-TF groups were compared via Student’s t-test. Linear regression analyzed associations between timing of TF, WLOS and MLOS.

**Results:**

Seventy-five patients (mean age 6.0 + 5.1 years) with a mean TBSA of 20.4 ± 4.4 %; 6.8 ± 8.4 % 3rd degree were included. Forty-four (n=33) patients received TF for a mean of 12.8±9.2 days (range = 1-35 days). The TF patients had significantly higher total and 3rd degree TBSA burn injuries (22.1 vs 19.0; p=0.002); 10.5 vs 4.0; p=0.0005 respectively) required more surgical procedures (3.4 vs 1.4; p< 0.000) and had a longer medical length of stay (24.9 vs 14.2 days; p< 0.000) versus the non-TF group. The TF patients with 20-30% TBSA burn injuries achieved a significantly larger percentage of their estimated protein needs, had a shorter WLOS (24.8 days vs 26.0 in non-TF; p=0.86) and experienced less weight loss from admission to discharge (-0.7% TF vs -2.9% loss in non-TF; p=0.22).

**Conclusions:**

Patients with 15-30% TBSA burn injuries often have difficulty meeting their increased nutritional needs with just an oral diet. Providing early supplemental enteral nutrition can enable the patient to reach their nutritional goals, minimize weight loss, and help shorten the time to full wound closure.

**Applicability of Research to Practice:**

Supplemental enteral nutrition support should be considered as an adjunct to an oral diet to meet the metabolic demands of pediatric patients with 15-30% TBSA burns.

**Funding for the Study:**

N/A

